# Screening of Long Non-coding RNAs Biomarkers for the Diagnosis of Tuberculosis and Preliminary Construction of a Clinical Diagnosis Model

**DOI:** 10.3389/fmicb.2022.774663

**Published:** 2022-03-03

**Authors:** Juli Chen, Lijuan Wu, Yanghua Lv, Tangyuheng Liu, Weihua Guo, Jiajia Song, Xuejiao Hu, Jing Li

**Affiliations:** ^1^Laboratory Medicine, Panzhihua Central Hospital, Panzhihua, China; ^2^Laboratory Medicine, Guangdong Provincial People’s Hospital, Guangdong Academy of Medical Sciences, Guangzhou, China; ^3^Laboratory Medicine, West China Hospital, Sichuan University, Chengdu, China

**Keywords:** long non-coding RNA, tuberculosis, molecular markers, machine learning algorithms, diagnostic models

## Abstract

**Background:**

Pathogenic testing for tuberculosis (TB) is not yet sufficient for early and differential clinical diagnosis; thus, we investigated the potential of screening long non-coding RNAs (lncRNAs) from human hosts and using machine learning (ML) algorithms combined with electronic health record (EHR) metrics to construct a diagnostic model.

**Methods:**

A total of 2,759 subjects were included in this study, including 12 in the primary screening cohort [7 TB patients and 5 healthy controls (HCs)] and 2,747 in the selection cohort (798 TB patients, 299 patients with non-TB lung disease, and 1,650 HCs). An Affymetrix HTA2.0 array and qRT-PCR were applied to screen new specific lncRNA markers for TB in individual nucleated cells from host peripheral blood. A ML algorithm was established to combine the patients’ EHR information and lncRNA data *via* logistic regression models and nomogram visualization to differentiate PTB from suspected patients of the selection cohort.

**Results:**

Two differentially expressed lncRNAs (TCONS_00001838 and n406498) were identified (*p* < 0.001) in the selection cohort. The optimal model was the “LncRNA + EHR” model, which included the above two lncRNAs and eight EHR parameters (age, hemoglobin, lymphocyte count, gamma interferon release test, weight loss, night sweats, polymorphic changes, and calcified foci on imaging). The best model was visualized by a nomogram and validated, and the accuracy of the “LncRNA + EHR” model was 0.79 (0.75–0.82), with a sensitivity of 0.81 (0.78–0.86), a specificity of 0.73 (0.64–0.79), and an area under the ROC curve (AUC) of 0.86. Furthermore, the nomogram showed good compliance in predicting the risk of TB and a higher net benefit than the “EHR” model for threshold probabilities of 0.2–1.

**Conclusion:**

LncRNAs TCONS_00001838 and n406498 have the potential to become new molecular markers for PTB, and the nomogram of “LncRNA + EHR” model is expected to be effective for the early clinical diagnosis of TB.

## Introduction

Tuberculosis (TB) is a major global infectious disease caused by *Mycobacterium tuberculosis* (MTB) infection that poses a serious risk to human health (World Health Organization [WHO], 2020). A diagnosis of TB relies heavily on laboratory tests. Existing laboratory tests for TB are mainly performed from pathogenic and host perspectives, including smear staining microscopy (Steingart et al., 2007; Parsons et al., 2011), culture, nucleic acid amplification test (Bautista-De Los Santos et al., 2016), *M. tuberculosis* gamma interferon release assay (TB-IGRA) (Ai et al., 2019), and purified protein derivative (PPD) test (Stavri et al., 2012). The accuracy and sensitivity of pathogenic tests are vulnerable to sample quality and sampling methods, which are not yet sufficient to explain the recent emergence of TB. Therefore, effective molecular markers and rapid and accurate strategies are urgently needed for early TB diagnosis.

Non-coding RNAs (ncRNAs) are produced during the transcription of the human genome into primary transcripts (Djebali et al., 2012), and they have greater tissue and spatiotemporal specificity than mRNAs and are involved in the body’s immune response and pathological damage processes in multiple ways (Distefano, 2018; Momen-Heravi and Bala, 2018). Host long non-coding RNAs (lncRNAs), a major subtype of ncRNAs, have potential as early molecular markers of TB. Studies have been conducted on host lncRNAs in TB patients, and the results showed that differentially expressed lncRNAs could affect the activation of T cells and helper T cells, resulting in a deficient immune response in TB patients (Chen et al., 2017). Other studies have shown that lncRNAs lnc-tgs1-1 and lnc-AC145676.2.1-6 are significantly downregulated in TB patients (Bai et al., 2019). However, the existing findings are insufficient in improving the early diagnosis of TB, and there is a need to screen for more lncRNA markers of TB specific to different populations.

In the process of clinical diagnosis and treatment, multi-item combination testing can significantly improve the diagnostic efficacy of the disease (Fang et al., 2021). Diagnostic models are the most common multi-indicator combination approach, and traditional diagnostic model studies (Gamil et al., 2018; Tu et al., 2020) are often statistical models based on laboratory indicators, which have limited data mining capabilities. In recent years, the use of machine learning (ML) (Baştanlar and Ozuysal, 2014) algorithms to construct multi-indicator combined diagnostic models of diseases with stronger data mining capability has become a hot spot for model research. The development and implementation of ML algorithms have made significant progress in recent years at the level of biomedical applications (Deo, 2015; Esteva et al., 2017; Yoon et al., 2017), especially in the field of medical image processing (Carin and Pencina, 2018; Mcbee et al., 2018). ML algorithm models for diseases based on images or laboratory metrics (Bogucki et al., 2019) and ML models for TB infection based on electronic health record (EHR) information have been reported (Thwaites et al., 2002).

The research of Taneja et al. (2017) and Li et al. (2018) of Stanford University showed that a combined model comprising EHR data and molecular markers was successful. The above studies suggest that the use of ML algorithms to integrate EHR data and molecular markers for combined modeling could make full use of the predictive value of molecular markers for TB, thereby greatly improving the diagnostic efficiency and clinical applicability of TB diagnostic models. This will hopefully lead to new breakthroughs for groups researching the pathogenesis of TB.

China is experiencing a severe TB epidemic, and the annual incidence (up to 695 cases per 100,000 people) is significantly higher in western China than in other regions of China (Wang et al., 2012). Therefore, based on previous research, this project investigated new TB-specific lncRNA molecular markers and explored their clinical diagnostic value. On this basis, ML algorithms were used to combine patient EHR information and lncRNA data to determine a differential diagnosis of TB to establish a model that can be visualized with a nomogram and to evaluate and validate the diagnostic efficacy and clinical applicability of the model, with the aim of providing a new direction for optimizing the clinical diagnosis of TB.

## Materials and Methods

**FIGURE 1:**
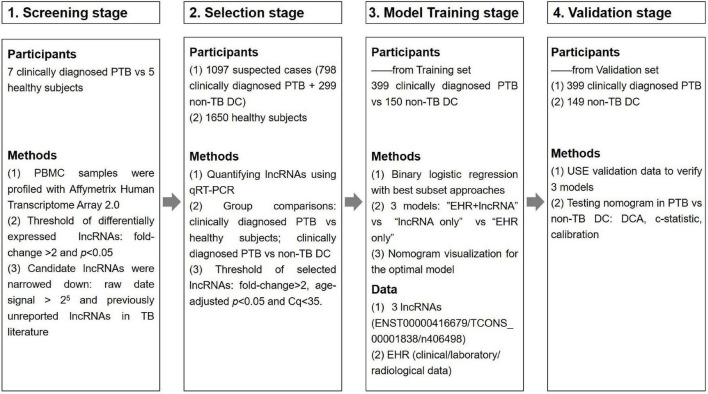
Overview of the strategy for investigating lncRNAs and prediction models for clinically diagnosed PTB patients.

This study was divided into four main phases: the primary screening stage, the selection stage, the modeling stage and the validation stage, as shown in Figure 1. Four candidate lncRNAs were selected from the HTA2.0 chip study between PTB patients and healthy subjects according to the criteria of “fold-change >2, original signal value >2^5^, and no previous reports in the literature (Mitchell et al., 2008)” in the primary screening stage. In the selection stage, qRT-PCR was used to detect the expression of candidate lncRNAs in a much larger selection cohort, and two lncRNAs were identified to be differentially expressed. In the model training stage, a binary logistic regression model for the differential diagnosis of TB was constructed by combining candidate lncRNAs with EHR data using patients with non-TB lung disease as a control; the optimal model was further visualized as a nomogram. Eventually, in the validation stage, an independent validation set was used to verify the three models and the nomogram’s differential diagnostic potential.

### Study Population and Information

From December 2014 to May 2019, 1,321 inpatients from the Tuberculosis Department and Respiratory Department of West China Hospital of Sichuan University were initially recruited, during which time 1,765 outpatient health examination personnel were also recruited. A TB diagnosis was made according to the WS 288-2017 Diagnostic Criteria for Tuberculosis (Ministry of Health, People’s Republic of China, 2008), and all cases were confirmed by two experienced respiratory physicians. Finally, a total of 2,759 (12 + 798 + 1,650 + 299) subjects were ultimately included in this study, as detailed in Table 1. The experimental procedures met the ethical requirements, and the study was authorized by the Ethics Committee of West China Hospital of Sichuan University [No. 2014(198)].

**TABLE 1:** Cohort distribution of PTB population study.

Queue name	TB patients (n)	Healthy controls (n)	Non-TB lung disease (n)
Primary screening cohort	7	5	/
Selection cohort	798	1650	299
Training set	399	/	150
Validation set	399	/	149

*In the modeling and verification stage, the tuberculosis patients and non-tuberculous lung disease patients in the selected cohort are randomly divided into training set and validation set.*

The inclusion criteria for patients with suspected PTB were as follows: (1) history of epidemiological exposure to TB; (2) signs and symptoms of active PTB at the time of consultation; (3) clear microbiological and radiological imaging evidence of PTB; and (4) effective response to anti-TB treatment. The exclusion criteria for patients with PTB were as follows: (1) lung diseases other than TB; (2) positive HIV, HBV or HCV serology; (3) history of chronic liver/renal/hematologic diseases or autoimmune diseases; (4) pregnancy; and (5) immune dysfunction or history of immunosuppressive therapy or booster agents.

The inclusion criteria for the 1,650 medical examination volunteers from the same period were as follows: no previous history of TB infection or negative MTB bacteriology (i.e., smear antacid staining, MTB culture, and TB-DNA) and confirmation as healthy upon physical examination.

The inclusion criteria for the patients with non-TB lung disease were as follows: (1) three consecutive negative MTB bacteriological tests (smear antacid staining, MTB culture, and TB-DNA examination); (2) clear diagnosis of a non-TB lung disease; and (3) effective antibiotic treatment or other non-TB drug regimens and confirmed by one year of follow-up observation. The 299 cases of non-TB lung diseases were dominated by pneumonia, pneumonia with other diseases, and lung cancer.

All imaging items and laboratory tests were performed at the Department of Imaging and Department of Laboratory Medicine of West China Hospital, respectively, and clinical tests and reports were completed by radiologists of West China Hospital who were unaware of the final diagnosis and grouping of patients.

### Long Non-coding RNAs Detection

RNA isolation and cDNA preparation: peripheral blood mononuclear cells (PBMCs) were isolated using DAKEWE human lymphocyte isolation tubes, and the total RNA from PBMCs was extracted by the TRIzol method. The concentration and purity of the extracted RNA were measured using a NanoDrop ND-1000 Microspectrophotometer, and RNA samples with absorbance ratios >1.8 at 260/280 nm and >2.0 at 260/230 nm were considered of acceptable quality. The integrity of the RNA samples was examined using agarose gel electrophoresis, and high-quality samples were retained for subsequent experiments. Complementary deoxyribonucleic acid (DNA) was prepared by removing genomic DNA contamination from the reaction system using the PrimeScript RT reagent kit.

An HTA2.0 microarray chip was used to detect the expression of lncRNAs in the primary screening cohort. The expression data of lncRNAs were analyzed, and Gene Ontology (GO) and pathway bioinformatics analyses were performed on the differential lncRNAs. Candidate lncRNAs were screened by the aforementioned criteria.

Total RNA from PBMCs of all subjects in the selection cohort was extracted, and the relative expression of candidate lncRNAs was detected by qRT-PCR. Relative lncRNA expression was measured in a blinded fashion, normalized to the endogenous control GAPDH, and calculated according to the 2^–ΔΔCq^ method (Mavridis et al., 2013), where ΔCq = Cq lncRNA − Cq GAPDH, ΔΔCq = ΔCq − Δaverage Cq (healthy subjects). The expression differences between TB patients and healthy controls (HCs) (TB vs. HC), TB patients and non-TB DC patients (TB vs. non-TB DC) were compared, and candidate lncRNAs with differences can be used as new molecular markers of tuberculosis and determined to be suitable for construction. Model target lncRNAs.

### Modeling

The patient population (TB patients and patients with non-TB lung disease) of the selection cohort was randomly divided into a training set (399 TB + 150 non-TB DC) and a validation set (399 TB + 149 non-TB DC). The training set was used for training the model, and the validation set was used for validating the model. Based on the lncRNA and EHR data in the training set, the R “bestglm” function was used for variable subset selection, and binary logistic regression was used to construct three models: “lncRNA + EHR,” “EHR,” and “lncRNA.” Their diagnostic performances were compared to determine the optimal differential diagnosis model for PTB.

Presentation and evaluation of the nomogram: the optimal model was visualized as a nomogram to assess its discrimination and calibration, and the nomogram was validated internally by applying a 500 bootstrap self-sampling method and externally validated with independent validation set data. The decision curve analysis (DCA) net benefit method was used to compare the clinical potentials of the nomograms and diagnostic models.

### Statistical Analysis

HyLown Power and Sample Size Calculators^1^ were used to estimate the required sample size of the lncRNA population study (Bacchetti and Leung, 2002) with the following parameters: α = 0.05, power value = 80%. Normally distributed count data were expressed as the mean ± standard deviation (X ± SD), and non-normally distributed count data were expressed using median and interquartile spacing [M (P25–P75)]. When comparing two groups, categorical variables were compared using the Chi-square test, and continuous variables were compared using the *t*-test or Mann–Whitney U test. Correlations between lncRNAs and clinical data were analyzed using the Spearman rank correlation test. All tests were two-sided probability tests, and *P*-values < 0.05 indicated a statistically significant difference. Data were analyzed, and the results were plotted using SPSS v24.0 and GraphPad Prism v8.0. Primary screening of modeling features (variables) was performed using R version 3.6.1 software and SPSS v24.0.

## Results

### Participant Information

There were no statistically significant differences in age or sex between TB patients and HCs in the primary screening cohort (7 males and 5 females, aged 22–59 years). The basic demographic information and laboratory indices of the selection cohort are detailed in Table 2, and 40 EHR indicators were collected for the modeling study. There were no statistically significant differences between the TB and HC groups matched for age, sex and body mass index (*P* > 0.05). The mean age of all patients in the disease control group (non-TB DC) was greater than that of the TB patients (*P* < 0.001). Significant differences were found in multiple laboratory tests, signs and symptoms, and imaging, suggesting that these indicators could be used as preliminary screening variables for modeling.

**TABLE 2:** Demographic and clinical features in Selection cohort.

Clinical feature	TB patients (*n* = 798)	Healthy controls *n* = 1650)	Adjusted-*P1*	Non-TB lung disease (*n* = 299)	Adjusted-*P2*
**Basic Information**					
Mean age(year) ± SD	40.81 ± 18.37	40.39 ± 12.73	0.659	57.02 ± 15.41	0.000
Gender (male/female, n)	444/354	874/776	0.214	182/117	0.132
Mean BMI (kg/m^2^) ± SD	21.59 ± 3.43	21.51 ± 3.52	0.843	21.11 ± 3.62	0.359
**Laboratory tests**					
Mean of erythrocytes (10^12^/L) ± SD	4.25 ± 0.77	4.91 ± 0.50	0.000	3.97 ± 0.85	0.000
Mean hemoglobin (g/L) ± SD	120.47 ± 23.23	145.59 ± 15.16	0.000	114.55 ± 25.34	0.000
Mean Hematocrit (%) ± SD	36.72 ± 6.61	44.93 ± 3.22	0.000	35.69 ± 7.16	0.031
Median of platelets (10^9^/L)(IQR)	233(167,311)	212(178,247)	0.000	208(145,294)	0.022
Median of ALT (U/L)(IQR)	17(11,31)	20(14,32)	0.007	21(15,37)	0.183
Median of AST (U/L)(IQR)	22(17,36)	20(16,26)	0.000	26(20,36)	0.097
Median of CRP (mg/L)(IQR)	21.80(6.21,53.37)	2.41(1.24,3.35)	0.000	12.00(5.15,38.50)	0.722
Median of ESR (mm/h)(IQR)	34(12,67)	13(2,16)	0.000	42(21,61)	0.314
Mean of albumin (g/L) ± SD	36.08 ± 6.67	48.69 ± 2.71	0.000	36.54 ± 6.49	0.306
Mean of globulin (g/L) ± SD	31.27 ± 6.98	27.55 ± 3.78	0.000	30.27 ± 7.71	0.040
Median of leukocytes (10^9^/L) (IQR)	6.06(4.65,8.30)	5.77(5.02,6.64)	0.000	6.67(4.86,9.07)	0.011
Median of lymphocytes (10^9^/L) (IQR)	1.08(0.75,1.51)	1.85(1.54,2.17)	0.000	1.29(0.90,1.81)	0.005
Median of neutrophils (10^9^/L) (IQR)	4.24(2.99,5.98)	3.38(2.80,4.07)	0.000	4.68(3.02,6.67)	0.113
Median of monocytes (×10^9^/L) (IQR)	0.46(0.32,0.64)	0.33(0.26,0.41)	0.000	0.43(0.30,0.65)	0.213
TB-IGRA positive (n,%)	357(44.74)	/	/	85(28.43)	0.000
**Symptoms**					
Cough (n,%)	434(54.38)	/	/	154(51.51)	0.433
Low fever (n,%)	413(51.75)	/	/	109(36.45)	0.000
Weight loss (n,%)	248(31.08)	/	/	40(13.38)	0.000
Night sweats (n,%)	313(39.22)	/	/	51(17.06)	0.000
Poor appetite (n,%)	336(42.10)	/	/	103(34.45)	0.021
Fatigue (n,%)	289(36.22)	/	/	124(41.48)	0.110
**Image inspection**					
polymorphic changes (n,%)	446(55.89)	/	/	103(34.45)	0.000
calcified foci (n,%)	136(17.04)	/	/	21(7.02)	0.000

*P-value was adjusted for age and gender between two groups; IQR, interquartile range; P1, P value for the comparison of TB cases and healthy controls (HCs) in the selection cohort; P2, P value for the comparison of TB cases and non-TB DCs (non-tuberculosis lung disease control patients) in the selection cohort.*

### Candidate Long Non-coding RNAs Selection

The quality control data of the microarray assays in the primary screening cohort of this study met the standards, indicating that the Affymetrix HTA 2.0 gene chip test was successful and can be used for lncRNA expression data analysis. A total of 325 differentially expressed lncRNAs were identified in the HTA 2.0 microarray between the TB and HC groups, of which 287 were upregulated and 38 were downregulated. According to the parameters fold-change >2, original signal value >2^5^, and no literature report available, the four candidate lncRNAs were three upregulated lncRNAs, namely, TCONS_00013664 (chr6:85677081–85678394), ENST00000416679 (chr7:26392–35472), and TCONS_00001838 (chr1:223963605–223971768), one downregulated lncRNA, n406498 (Chr2:113599027–113601576).

### Bioinformatics Analysis of Candidate Long Non-coding RNAs

**FIGURE 2:**
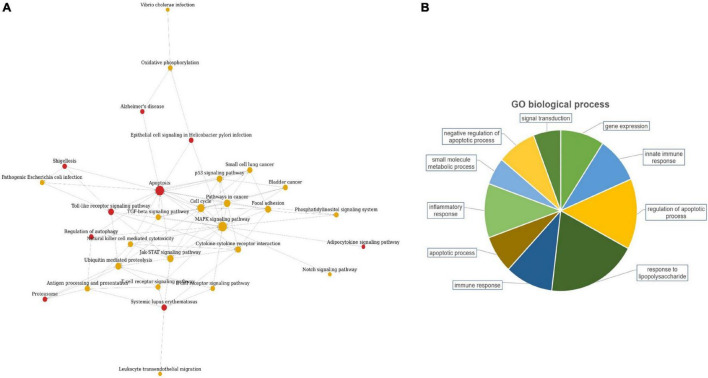
Bioinformatics analysis of candidate lncRNAs.

The candidate lncRNAs were further bioinformatically analyzed to predict the signal transduction pathways and biological processes that might be associated with them, and the results are shown in Figure 2. Pathway analysis showed a total of 30 enriched signaling pathways for the differentially expressed lncRNAs, of which the top 10 enriched scores were mainly associated with the MAPK signaling pathway, apoptosis, cell cycle, and the Jak-STAT signaling pathway. GO analysis showed that the differentially expressed lncRNAs may be involved in regulating numerous biological processes, such as lipopolysaccharide response, apoptosis, inflammatory response, immune response, and small molecule metabolism.

### Differentially Expressed Candidate Long Non-coding RNAs in the Selection Cohort

**FIGURE 3:**
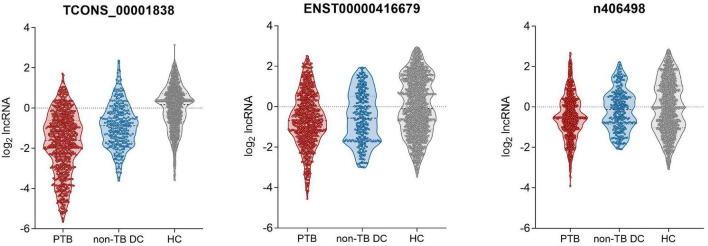
Expression of candidate LncRNAs in selection cohort.

qRT-PCR was used to detect the expression of the four candidate lncRNAs in the selection cohort of TB patients and HCs. It was found that lncRNA TCONS_00013664 had extremely low expression (Cq > 35) in both groups and was excluded from further analysis (Bustin et al., 2009). The relative expression levels of the remaining three lncRNAs are shown in Figure 3 and Table 3. The relative expression levels of ENST00000416679, TCONS_00001838, and n406498 in the HC group were 0.94, 1.12, and 0.99, in the TB group were 0.62, 0.33, and 0.70, and in the non-TB DC group were 0.68, 0.54, and 0.91, respectively. The expression of the three lncRNAs was significantly different between the TB and HC groups (all *P* < 0.001). The area under the ROC curves (AUCs) of the three lncRNAs were all >0.50, the AUC of lncRNA TCONS_00001838 was 0.828, the sensitivity was 76.24%, and the specificity was 72.93%. The AUC of lncRNA ENST00000416679 was 0.622, the sensitivity was 38.24%, and the specificity was 81.45%. The AUC of lncRNA n406498 was 0.602, the sensitivity was 37.88%, and the specificity was 82.58%, the results are shown in Supplementary Table 1. The expression of TCONS_00001838 and n406498 was significantly different between the TB and non-TB DC groups (*P* < 0.001). Suggesting that TCONS_00001838 and n406498 could be used as preliminary screening variables for modeling.

**TABLE 3:** lncRNA expression in Selection cohort (2^–ΔΔCq^).

LncRNA	Healthy controls (*n* = 1650)	TB patients (*n* = 798)	Adjusted-*P1*	Non-TB lung disease (*n* = 299)	Adjusted-*P2*
*ENST00000416679*	0.94 (0.46–2.39)	0.62 (0.36–1.25)	<0.001	0.68 (0.32–1.44)	0.367
*TCONS_00001838*	1.12 (0.62–1.65)	0.33 (0.14–0.65)	<0.001	0.54 (0.32–0.88)	<0.001
*n406498*	0.99 (0.49–2.07)	0.70 (0.46–1.17)	<0.001	0.91 (0.55–1.44)	<0.001

*P-value was adjusted for age and gender between two groups, IQR, interquartile range; P1, P value for the comparison of TB cases and healthy controls (HCs) in the selection cohort; P2, P value for the comparison of TB cases and non-TB DCs (non-tuberculosis lung disease control patients) in the selection cohort.*

### Diagnostic Modeling and Nomogram Visualization

To establish a diagnostic model for distinguishing tuberculosis patients from non-tuberculous lung disease patients, 12 characteristic variables were initially included in this study for model construction: the 2 lncRNA loci and 10 EHR indicators mentioned above (age, hemoglobin, white blood cell count, lymphocyte count, gamma interferon release test, hypothermia, weight loss, night sweats, imaging examinations showing polymorphic changes, and calcified foci). Three binary logistic regression models were constructed: an “LncRNA + EHR” combined model, “LncRNA” model and “EHR” model. The optimal subset of variables included in each model was evaluated by the exhaustive search method. Finally, the above two lncRNA loci and eight EHR indicators (age, hemoglobin, lymphocyte count, gamma interferon release test, weight loss, night sweats, imaging examinations showing polymorphic changes, and calcified foci) were selected as optimal subset of variables. The variance inflation factors of the characteristic variables ranged from 1.06 to 1.19, indicating that there was no covariance among the indicators included in the models.

**FIGURE 4:**
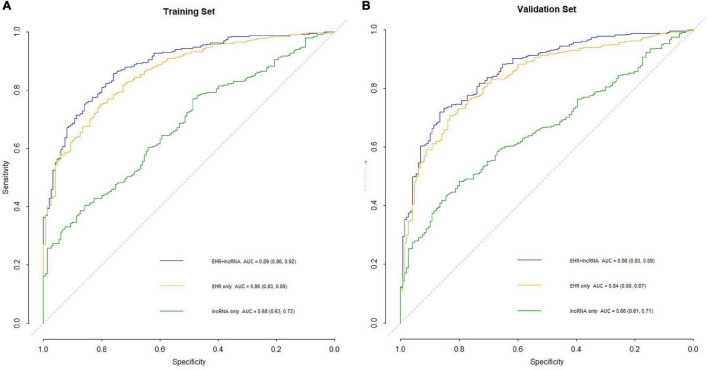
ROC curves of the three models in selection cohort.

ROC curves of the three models in the selection cohort are shown in Figure 4. In the training set, the AUCs of the three models are shown in Table 4. The AUC of the “LncRNA + EHR” model was 0.89, the highest among the three models which was significantly higher than that of the “LncRNA” and “EHR” models (*P* < 0.001), For the differential diagnosis of TB, the “LncRNA + EHR” model had a sensitivity of 0.86 and a specificity of 0.76, This shows that the optimal model for the differential diagnosis of PTB was the “LncRNA + EHR” model. The likelihood ratio test, McFadden *R*^2^ test and Nagelkerke *R*^2^ test were used to assess the goodness of fit of the “LncRNA + EHR” model with 10 variables, and the results were *P* < 0.0001, 0.372, and 0.5, respectively. Indicating that the model variables have good goodness of fit and that there is no overfitting.

**TABLE 4:** AUC of the three diagnostic models in the training set.

Model	AUC(95%CI)	Z value	*P*
“LncRNA+EHR” model	0.89(0.86–0.92)		
“EHR” model	0.86(0.83–0.89)	3.224	0.001*
“LncRNA”model	0.68(0.63–0.72)	9.081	<0.001**

The test method is DeLong’s test, *: the comparison between the “EHR” model and the “LncRNA+EHR” model; **: the comparison between the “LncRNA” model and the “LncRNA+EHR” model.

**FIGURE 5:**
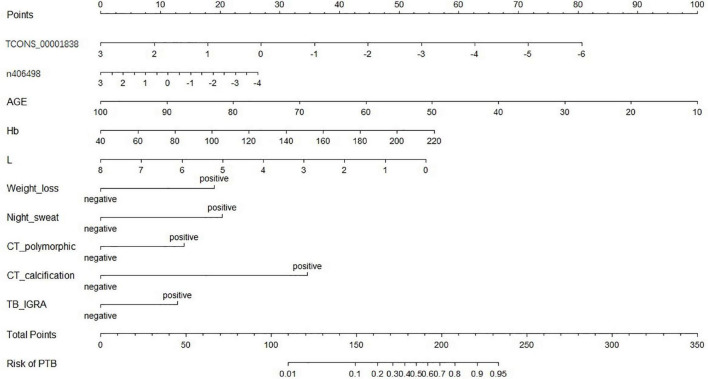
Nomogram of the optimal model (“lncRNA + EHR” model).

A nomogram of the visualized “LncRNA + EHR” model is shown in Figure 5, and the distribution of the scores and weights of the variables in the nomogram showed that TCONS_00001838 (lncTP), age (AGE), and lymphocyte count (L) were all negatively correlated. In contrast, ENST00000416679 (lncPA), hemoglobin (Hb), weight loss (weight loss), night sweating (Night_sweat), imaging showing polymorphic changes (CT_polymorphic), imaging showing calcification (CT_calcification), and gamma interferon release test (TB_IGRA) were the positively correlated variables.

### Validation the Nomogram

**FIGURE 6:**
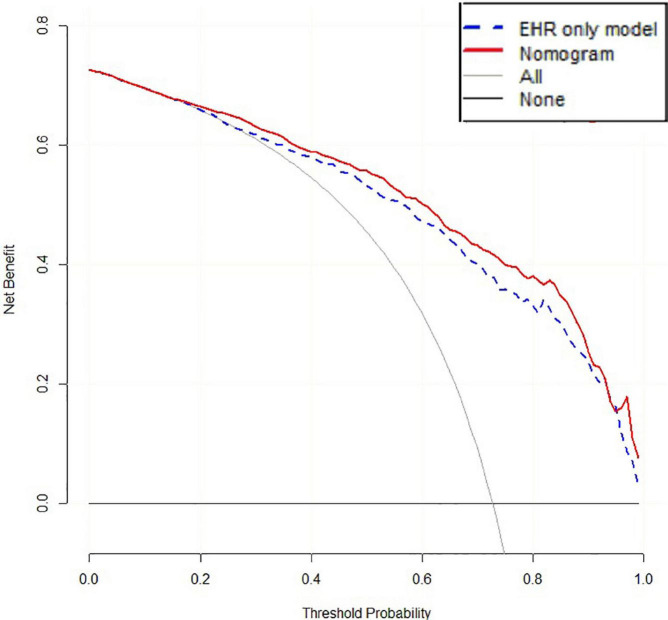
DCA of the “EHR” model and nomogram.

The accuracy of 500 “bootstrap” self-sampling validations using the training set data was 0.83 (0.80–0.86), and the calibration curve and Hosmer–Lemeshow test were not significantly different (*P* = 0.755), confirming the high consistency between the predicted results of the nomogram and the actual results. Using the validation set data to validate the three models, the “LncRNA + EHR” model also had the best diagnostic performance for the differential diagnosis of TB, with an accuracy of 0.79 (0.75–0.82), a sensitivity of 0.81 (0.78–0.86), a specificity of 0.73 (0.64–0.79), and an AUC of 0.86. The calibration curve and the Hosmer–Lemeshow test (*P* = 0.174) showed that the nomogram also showed good predictive compliance for the differential diagnosis of TB in the validation set. Using DCA to compare the potential clinical application of the combined model with the conventional “EHR” model, the results of the DCA analysis in Figure 6 show that at a threshold of 0.2–1, the nomogram (blue dashed line) is more accurate than the conventional “EHR” model (red line). This shows that the use of the nomogram may improve the prediction level of PTB, indicating that the nomogram has better predictive value for clinical application.

## Discussion

In our study, the expression levels of lncRNA ENST00000416679, TCONS_00001838, and n406498 in tuberculosis patients in western China were significantly downregulated compared with those in healthy people (*P* < 0.001). The expression levels of lncRNA TCONS_00001838 and n406498 were also significantly different between the tuberculosis patients and non-TB lung disease groups (*P* < 0.001). As of 30 June 2021, none of the above three lncRNAs has been reported in the literature. Therefore, this is the first study to determine that these three lncRNAs may be related to the development of tuberculosis. LncRNAs TCONS_00001838 and n406498 may become new molecular markers of tuberculosis. More importantly, we developed and verified a new nomogram containing lncRNA features and conventional EHR features that can effectively distinguish PTB patients from patients with suspected diseases.

A number of studies have shown that lncRNAs can be used as molecular biomarkers for disease diagnosis and prognosis. For example, the expression level of serum lncRNA UCA1 is very important for the early diagnosis of gastric cancer (Gao et al., 2015). The abnormal expression of lncRNAs can affect not only the pathological process of TB but also lncRNA polymorphisms (which can also affect the susceptibility of individuals to TB), suggesting that lncRNAs have a profound impact on the pathogenesis of TB (Zhao et al., 2017). In our study, the expression levels of TCONS_00001838 and n406498 were significantly downregulated in TB patients in western China (*P* < 0.001) and lower than those of healthy people. The AUC was further used to assess the diagnostic value of the two lncRNAs in TB, which all had AUCs >0.50; the AUC of lncRNA TCONS_00001838 was highest at 0.828, with a sensitivity of 76.24% and a specificity of 72.93%. Compared with the common host diagnostic indicators of TB in widespread clinical use today, the diagnostic efficacy of lncRNA TCONS_00001838 for TB was close to that of TB-IGRA (Wang et al., 2018) (AUC = 0.860, sensitivity 91.67%, specificity 74%) and significantly better than that of the TB antibody test (AUC = 0.691, sensitivity 45.1%, specificity 93.1%) and PPD test (AUC = 0.738, sensitivity 72.1%, specificity 75.5%) (Gong, 2019), which are all used for clinical diagnosis. Based on the above research results, we believe that TCONS_00001838 and n406498 have the potential to become new molecular markers of PTB and could serve as protective factors against MTB infection.

The specific functions of the two target lncRNAs we discovered (TCONS_00001838 and n406498) and their mechanism of action related to TB are still unclear. To explore the possible regulatory mechanisms of the three lncRNAs, we conducted pathway and GO analyses using gene chips. The results of pathway analysis indicated that the differentially expressed lncRNAs were mainly associated with the MAPK signaling pathway, apoptosis, cell cycle, Jak-STAT signaling pathway and Toll-like receptor signaling pathway. The results of GO analysis showed that the differentially expressed lncRNAs may be involved in regulating numerous biological processes, such as lipopolysaccharide response, apoptosis, inflammatory response, immune response, and small molecule metabolism. This is consistent with previous reports on the prediction of lncRNA studies in TB (Lee et al., 2018; Shukla et al., 2018; Li et al., 2019). Atianand et al. (2016) reported that a lncRNA gene that can promote the survival of red blood cells can also inhibit the uncontrolled inflammatory response in macrophages. Yi et al. (2014) used gene chip technology to identify a large number of differentially expressed lncRNAs and mRNAs in CD4+ T lymphocytes in the peripheral blood from patients with TB infection and from HCs, indicating that the lncRNAs and mRNAs of CD4+ T lymphocytes may be involved in the occurrence and development of TB. Based on the above analysis, we speculate that the possible mechanisms of action of lncRNAs TCONS_00001838 and n406498 are related to the immune response involved in the regulation of macrophages and lymphocytes or to the regulation of TB infection by lymphocytes. However, there are some limitations in this study: the regulatory mechanisms of TB infection were not investigated for the TCONS_00001838 and n406498 genes; these need to be explored in depth and functionally validated.

In recent years, ML algorithms have gradually been applied to the medical field, integrating multidimensional information such as genetic data and clinical data, which is conducive to achieving breakthrough discoveries in the research of disease mechanisms and molecular markers (Trakadis et al., 2019). We used two lncRNAs—TCONS_00001838 and n406498—as the target lncRNAs of the diagnostic model and incorporated eight EHR indicators to construct a combined diagnostic model. When the lncRNAs and clinical EHR information were combine modeled, they could effectively compensate for a lack of EHR information and had obvious advantages in the differential diagnosis of PTB. The diagnostic efficacy of the “LncRNA + EHR” model was significantly higher than that of the EHR model and had better reliability. At the same time, the EHR variables included in the “LncRNA + EHR” model included some traditional TB predictors (Pinto et al., 2013), which had better clinical interpretability. The nomogram can be used to judge or predict the occurrence or progression of diseases and provide personalized quantitative risk indicators for disease diagnosis and prognosis assessment. Visualization of the nomogram of the “LncRNA + EHR” model is expected to be a valuable clinical diagnostic tool for PTB. This study proves that the nomogram has higher net benefits than the “EHR” model through the analysis of the decision curve, and it has more advantages. The good c-statistic, calibration and DCA results of the nomogram show that adding lncRNAs to the EHR model can not only improve the accuracy of the model for identifying tuberculosis but also increase the clinical application value of the traditional model, but the specific effect should also be tested by clinical practice and evidence-based medicine.

## Conclusion

In summary, this study is the first to propose that the expression of lncRNAs TCONS_00001838 and n406498 is associated with tuberculosis and may be a potential molecular biomarker for the early diagnosis of tuberculosis. The nomogram of “LncRNA + EHR” model is expected to become an effective tool to assist the clinical diagnosis of TB.

## Data Availability Statement

Publicly available datasets were analyzed in this study. This data can be found here: the microarray data have been deposited in the Gene Expression Omnibus (GEO) under the accession GSE119143.

## Author Contributions

JC and LW wrote the main manuscript text and participated in the experiment all the way. XH and JL participated in modifying the manuscript and designed the study. YL and WG participated in the analysis of data and prepared tables and figures. TL and JS engaged in the acquisition of data (laboratory or clinical). All authors have reviewed the manuscript.

## Conflict of Interest

The authors declare that the research was conducted in the absence of any commercial or financial relationships that could be construed as a potential conflict of interest.

## Publisher’s Note

All claims expressed in this article are solely those of the authors and do not necessarily represent those of their affiliated organizations, or those of the publisher, the editors and the reviewers. Any product that may be evaluated in this article, or claim that may be made by its manufacturer, is not guaranteed or endorsed by the publisher.

## Footnotes

1
http://powerandsamplesize.com/Calculators/
