# Supplementary material for: Screening of Long Non-coding RNAs Biomarkers for the Diagnosis of Tuberculosis and Preliminary Construction of a Clinical Diagnosis Model
Source: Front Microbiol. 2022 Mar 3;13:774663. doi: 10.3389/fmicb.2022.774663 (PMC8928272; doi:10.3389/fmicb.2022.774663)
Supplement: Supplementary file 1 [file Table_1.docx]

Supplementary Table 1 The diagnostic efficacy of three LncRNAs in this study

| LncRNA | AUC  (95%CI) | sensitivity ^*^  %(95%CI) | specificity ^*^  %(95%CI) | *P*值 |
| --- | --- | --- | --- | --- |
| *ENST00000416679* | 0.622  (0.599-0.645) | 38.24  （35.93-40.61） | 81.45  （78.61-84.00） | <0.001 |
| *TCONS_00001838* | 0.828  (0.810-0.845) | 76.24  (74.13-78.23) | 72.93  (69.75-75.90) | <0.001 |
| *n406498* | 0.602  (0.579-0.624) | 37.88  (35.57-40.24) | 82.58  (79.80-85.06) | <0.001 |

Note: * refers to the sensitivity and specificity at the maximum Youden index.
